# Integrated genomic and BMI analysis for type 2 diabetes risk assessment

**DOI:** 10.3389/fgene.2015.00075

**Published:** 2015-03-17

**Authors:** Dayanara Lebrón-Aldea, Emily J. Dhurandhar, Paulino Pérez-Rodríguez, Yann C. Klimentidis, Hemant K. Tiwari, Ana I. Vazquez

**Affiliations:** ^1^Institute of Mathematics, School of Science and Technology, Universidad MetropolitanaSan Juan, Puerto Rico; ^2^Department of Health Behavior, School of Public Health, University of Alabama at BirminghamBirmingham, AL, USA; ^3^Department of Statistics, Colegio de PostgraduadosTexcoco, México; ^4^Division of Epidemiology and Biostatistics, Mel and Enid Zuckerman College of Public Health, University of ArizonaTucson, AZ, USA; ^5^Department of Biostatistics, School of Public Health, University of Alabama at BirminghamBirmingham, AL, USA

**Keywords:** type 2 diabetes, Logistic Regression, Neural Network, risk assessment, genetic score

## Abstract

Type 2 Diabetes (T2D) is a chronic disease arising from the development of insulin absence or resistance within the body, and a complex interplay of environmental and genetic factors. The incidence of T2D has increased throughout the last few decades, together with the occurrence of the obesity epidemic. The consideration of variants identified by Genome Wide Association Studies (GWAS) into risk assessment models for T2D could aid in the identification of at-risk patients who could benefit from preventive medicine. In this study, we build several risk assessment models, evaluated with two different classification approaches (Logistic Regression and Neural Networks), to measure the effect of including genetic information in the prediction of T2D. We used data from to the Original and the Offspring cohorts of the Framingham Heart Study, which provides phenotypic and genetic information for 5245 subjects (4306 controls and 939 cases). Models were built by using several covariates: gender, exposure time, cohort, body mass index (BMI), and 65 SNPs associated to T2D. We fitted Logistic Regressions and Bayesian Regularized Neural Networks and then assessed their predictive ability by using a ten-fold cross validation. We found that the inclusion of genetic information into the risk assessment models increased the predictive ability by 2%, when compared to the baseline model. Furthermore, the models that included BMI at the onset of diabetes as a possible effector, gave an improvement of 6% in the area under the curve derived from the ROC analysis. The highest AUC achieved (0.75) belonged to the model that included BMI, and a genetic score based on the 65 established T2D-associated SNPs. Finally, the inclusion of SNPs and BMI raised predictive ability in all models as expected; however, results from the AUC in Neural Networks and Logistic Regression did not differ significantly in their prediction accuracy.

## Introduction

Type 2 Diabetes (T2D) is one of the fastest growing diseases in the United States and other developed nations (Nugent, 2008; Hu, 2011). In the last three decades, the number of Americans diagnosed with diabetes has tripled (from 5.6 to 20.9 million), making this a public health concern (CDC (Center for Disease Control), 2013). T2D is a chronic metabolic disease, characterized by high levels of glucose in the blood, and frequently caused by a deficiency of insulin secretion and/or the development of insulin resistance (the inability of cells to respond to the insulin). If not treated properly, it can produce kidney failure, blindness, and circulatory problems. (Manzella, 2007; Buijsse et al., 2011; Hu, 2011; Sanghera and Blackett, 2012). The interplay of environmental (i.e., sedentary life, obesity, lack of exercise, poor diet) and genetic factors (i.e., familial contribution), contribute to the etiology and epidemy of T2D, in addition to an estimated heritability of 26% (Poulsen et al., 1999). Since 2007, Genome Wide Association Studies known as GWAS, have identified and confirmed more than 50 loci associated with the development of T2D (Steinthorsdottir et al., 2007; Lindgren et al., 2009; Shu et al., 2010; Voight et al., 2010; Morris et al., 2012). Several genes identified so far are involved in encoding proteins necessary for insulin secretion, glucose metabolism, and beta-cell function, which are components that enable insulin production and insulin receptor activation in the body (Sladek et al., 2007; Steinthorsdottir et al., 2007; Yasuda et al., 2008).

Previous studies that have included genetic profiling and scores in T2D preventive models, have shown only a slight increase in predictive ability. Generally, the use of genetic variants provides a small contribution in terms of prediction accuracy due to their small effects, especially if compared to the use of age and clinically measured variables, such as BMI, and triglyceride levels and known risk factors for this disease (Saxena et al., 2007; Lyssenko et al., 2008; Voight et al., 2010; Vazquez et al., 2012). As of today, while there is excitement with the possibility of a more personalized medicine, medical professionals do not consider genotypic information as a variable in assessing patients' risk of developing T2D (Katsios, 2010; Lyssenko and Laakso, 2013). In several studies where risk assessment models have been built and tested, a few deficiencies have been noticed that could possibly have influenced their models' predictive ability. Such deficiency may arise due to the use of a model that so far does not capture the complexity of polygenic signals and their interaction with covariates. In addition, an ideal risk assessment model would incorporate the interplay of a substantial number of small-effect genes and several phenotypic variables (e.g., BMI) related to the development of T2D in order to get a more realistic and precise prediction (Lindstrom and Tuomilehto, 2003). However, by incorporating other phenotypes (also heritable) into the risk assessment models, pleiotropic genetic effects shared by both traits could be explained. BMI is an easy to measure phenotype, highly associated to diabetes and obesity and shown to be a strong predictor of diabetes (Lyssenko et al., 2008; Meigs et al., 2008). Nevertheless, it is possible that after accounting for BMI, the inclusion of SNP variants associated to T2D, may not improve prediction accuracy any further. However, this is an unanswered question.

To address these problems, we applied two statistical models (logistic regression, and a neural network) to data from the Framingham Heart Study, and incorporated 65 SNPs that are confirmed to be associated with T2D (Morris et al., 2012) to estimate genetic and non-genetic effects in the prediction of T2D. Since non-genetic factors play a predominant role in whether genetically predisposed individuals progress on to T2D (Poulsen et al., 1999), we considered including BMI information at the onset of T2D, and importantly including genetic by BMI interactions in the predictions of T2D.

## Materials and methods

### Data

Our data set (*n* = 5239) came from the Framingham Heart Study which followed participants over seven decades and collected information from bi-yearly physical and blood examinations. Our sample was composed of 2378 females and 2861 males from the Original and Offspring cohorts; where 4300 are controls and 939 subjects are cases. Diagnosis of T2D for subjects varied by cohort. In the Original cohort, the presence of T2D was diagnosed with a blood glucose level greater than or equal to 200 mg/dL; however, for the offspring cohort, diabetes was diagnosed if fasting glucose levels were equal or greater to 125 mg/dL (NCBI, 2006, 2008).

We also examined 65 SNPs that were found to be associated with T2D as listed in Morris et al. (2012). Since only 20 of the 65 SNPs were genotyped by the Affymetrix 500K chip in our sample, genotype imputation was performed for the missing genotypes of the SNPs by using the IMPUTE2 software (Howie et al., 2011). Missing information per SNP was imputed with a mean accuracy of 0.94. The imputation accuracy for all the imputed SNPs can be seen in Table A in Supplementary Materials.

### Models

In this section we will present the response variable, the set of predictors, and the genetic covariates used to build the T2D models. Subsequently, the parametric and non-parametric methods, Logistic Regression (LR) and Neural Network (NN), respectively, will be introduced and finally, we will detail a series of nested models that incorporate BMI and genetic components consisting of the 65 SNPs (Morris et al., 2012).

#### Set of response and predictor variables

Disease status of the participants was coded with a binary response variable *y*(*y*_*i*_ = 0 for absence and *y*_*i*_ = 1 for presence of T2D in the *i^th^* subject). A group of covariates was selected based on the association with T2D (*P* < 0.01) and these were: cohort (*c*_*i*_), a dummy variable indicating whether the subject *i* belongs to the Original or Offspring cohort; age at last contact (*l*_*i*_) 73.91 ± 11.74 (mean ± s.d.), was included to control for different exposure time or observational period; the first two principal components (*PC*_1_, *PC*_2_) derived from a set of 1000 European ethnicity-informative SNPs (Drineas et al., 2010), and gender (*s*_*i*_), also coded with an indicator variable, with this set of co-variables we generated a baseline model that is not influenced by genetic effects. Each one of the risk assessment models was extended by incorporating the body mass index (BMI, *b*_*i*_) at diabetes onset in the case of diabetics and the last observed BMI for non-diabetics, which served as a measure of obesity [*b*_*i*_ (*mean* ± *s.d.*) = 27.75 ± 5.38]. In some models, the SNPs were incorporated either by directly including the 65 SNPs or indirectly by a genetic score (GS) calculated as the count of risk alleles presents on each subject per SNP $\left(GS_{i}=\sum_{j=1}^{65}x_{ij};x_{ij}=\{0,\;1,\;2\}\right)$. Where *x*_*ij*_ are the count of risk alleles in the *j^th^* SNP for the *i^th^* subject. Risk alleles for the inputted SNPs were given by the expected allele count *x*_*ij*_ being this a continuous number ranging from [0, 2].

#### Logistic regression

The probability of diabetes peculiar to subject *i^th^* was given by a linear predictor with a logit link (Dobson, 2002) in the following form:

(1) $$p_{i}=E\left(y_{i}|\cdot \right)=\frac{\exp\left(\eta_{i}\right)}{1+\text{exp}(\eta_{i})}$$

where *E*(*y*_*i*_|·) is the expected value for the diabetes status (*y*_*i*_); *p_*i*_ is* the subject-specific probability of developing T2D given a set of covariates for subject *i* and *exp*(·) is the exponential function. The linear predictor (η_*i*_) for a model built with only the non-genetic predictor variables is described in equation (2) and obtained as follows:

(2) $$\eta_{i}=\alpha_{0}+\alpha_{1}c_{i}+\alpha_{2}s_{i}+\alpha_{3}b_{i}+\alpha_{4}l_{i}$$

where α_0_ is an intercept common to all observations, plus a regression on the “fixed effects”; and α_1_ to α_4_ are the corresponding regression coefficients or effects, for each one of the included variables.

#### Neural network

Bayesian Regularized Neural Network is a machine learning algorithm that is suited for classification problems (Shekhar and Amin, 1992; Neal, 1996; Gianola et al., 2011; Pérez-Rodríguez et al., 2012). The Neural network aims to reduce the errors in the training set, adjust the parameters and to respond properly to novel inputs. One of the simplest neural networks is composed of three layers: the input layer which consists of the input of all the covariates for each one of the subject's *x*_*ij*_ (*i* = 1… 5245; *j is the quantity of covariates included per model)* the hidden layer that contains *s* neurons; and the output layer. Each input connects to each one of the neurons creating an unknown weight *w_*i*_* for each input. This inner product between the weights and the input vector in each neuron of the hidden layer is given by equation:

(3) $$u_{ki}=b_{0}+\sum_{j\;=\;1}^{65}\beta_{jk}x_{ij},k=1,\ldots ,s\left(\text{neurons}\right),$$

where *u*_*ki*_ in the hidden layer is transformed by applying an activation function. We used the tangent hyperbolic function: $g\left(a\right)=\frac{\exp\left(2a\right)-1}{\exp\left(2a\right)+1}$, which maps the inputs into the closed interval [−1, 1]. The output from each of the neurons is combined linearly $z_{i}=\mu +\sum_{k=1}^{s}w_{k}g(u_{ki})$ and finally transformed by applying the function $h\left(a\right)=\frac{1}{1+exp\left(-a\right)}$, which maps the inputs into an open interval (0, 1), so that the output can be interpreted as a probability, that is *y*_*i*_ = *h*(*z*_*i*_). Since the activation function can be a nonlinear function, it allows the classifier to capture non-linear effects.

Neural network models were fitted using the Bayesian approach (MacKay, 1992) implemented in the Software for Flexible Bayesian Modeling (FBM) written by Neal (1996) which is available freely at www.cs.toronto.edu/~radford/fbm.software.html. For our analyses, a total of 6 neurons were included in the hidden layer to reduce the computational burden, since the results with 9 neurons yielded almost identical results.

#### Sequence of models

Six models were built, with the aim of evaluating the genetic effects of the 65 variants associated to T2D as risk factors. Our starting point was a Baseline model (BASE), which is composed of only the non-genetic covariates or fixed effects: cohort, age at last contact, gender and principal components. BASE_BMI_ extends model BASE by incorporating BMI in the set of predictors. Since BMI co-varies with T2D, is reasonable to think that pleiotropic effects may exist. Subsequently, we generated clinical models that included genetic information. GEN65 extends BASE by incorporating the 65 SNPs associated to T2D; each SNP contains the count of risk alleles {0, 1, 2}. The GENS extends BASE model by adding the Genetic Risk Score (GS) consisting of the sum of all variants that increase diabetes risk. To test whether there are genetic effects on T2D after accounting for BMI, models GENS_BMI_ and GEN_BMI_ are extensions of the model of GENS and GEN65, respectively, including BMI. Finally, GEN_BMI_ was also extended accommodating SNPs by BMI interactions, into a model called GENB_SNPs × BMI_. Table 1, shows the components inside of each one of the models tested.

**Table 1:** **Description of the model's components**.

**Model components**				
**Model name**	**Covariates (age, gender, PCs, cohort and exposure time)**	**BMI**	**65 SNPs**	**Genetic score**
BASE	✓			
BASE_BMI_	✓	✓		
GEN65	✓		✓	
GEN65_BMI_	✓	✓	✓	
GENS	✓			✓
GENS_BMI_	✓	✓		✓

#### Estimated effects and confidence intervals

The estimated effects of gene markers and other covariates for the risk of T2D were calculated and displayed in terms of Odds Ratio (OR). The BASE model was used to estimate the effects for all the non-genetic covariates. In addition *P*-values were used to discriminate SNPs association to T2D and a 95% Confidence Interval of the OR was built to determine the statistical significance of the association between the response and the predictors.

### Predictive ability

To evaluate the risk assessment models, a 10-fold cross-validation was used to compare the accuracy of their respective predictions. Each of the subjects within the data was assigned randomly to the 10 folds. The testing sample consisted of a subset of 1/10th of the data, and training would take the rest of the sample in order to achieve an optimal predictive model. Predictive ability of the models was assessed with the Receiver Operating Characteristic Curve (Fawcett, 2006), using the R package “pROC” (Robin et al., 2013), in order to obtain their Area Under a Curve (AUC), also referred as C-Statistic.

## Results

### Descriptive statistics

The characteristics of the 5245 subjects are described and summarized in Table 2. More than half of the sample were females (*n* = 2864), and only 18% of the overall subjects were diabetic. Within the data set, BMI (mean ± standard deviation) for diabetics was 29.9 ± 6.0, and healthy subjects 27.3 ± 5.1. According to the subjects BMI indexes, 28.2% of the observed subjects demonstrated to be obese (*n* = 1482) and 67.4% of the sample were overweight, while the rest were classified as normal. The mean observed age at which sample subjects acquired T2D was 63 years old. A reduction in the proportion of incidences of T2D can be seen in the Offspring cohort since the subjects of the Original cohort were observed during a longer time when compared to the Offspring cohort.

**Table 2:** Descriptive statistics of the sample (*n* = 5245)^*^.

**Covariates**	**Diabetics**	**Non-diabetics**
Original Cohort (*n* = 1497)	30.2% (452)	69.8% (1045)
Offspring Cohort (*n* = 3742)	13.0% (487)	87% (3255)
Males	20.6% (489)	79.5% (1892)
Females	15.7% (450)	84.3% (2414)
BMI (mean ± s.d.)	29.9 ± 5.9	27.3 ± 5.1
Exposure Time (mean ± s.d.)	78.8 ± 10.6	72.9 ± 11.8

* Frequency of subjects per division are enclosed between parenthesis (n).

### Genetic score

GS is a subject specific count of all the risk alleles in each one of the SNPs reported to be associated with risk of T2D. Table 3 shows a summary of the GS for both control and cases. GS ranged from 52 to 86, which indicates that each individual had at least one risk allele for T2D in almost every SNP. Individuals with a high genetic score presented a greater cumulative incidence of T2D, in comparison to subjects with a low risk score.

**Table 3:** **Genetic score frequencies per quartile**.

**Genetic Score**	**Frequencies by diabetes status**	
**Quartiles**	**Non-diabetic, percentage (*n*)**	**Diabetics, percentage (*n*)**
< 66.32	86% (1132)	14% (182)
66.32 ≤ GS < 69.55	85% (1108)	15% (199)
69.55≤ GS < 72.75	82% (1072)	18% (236)
≥72.75	75% (992)	25% (322)

### Estimated effects

NN is a classifier that yields multiple estimated effects (depending on the number of neurons), which complicates the interpretation of the results. For that reason, estimates shown in this section are results from the Logistic Regression model.

Table 4 shows the estimated Odds Ratio for the significant covariates in all models. If these covariates are not augmenting T2D risk, we would expect an OR estimate and both limits of the 95% confidence interval to include 1.0. All covariates except the Principal Components were significantly associated to diabetes (*P* < 0.01). Fixed effects estimates across the models were consistent for each of the covariates (i.e., the inclusion or exclusion of effects in the model produced very little variation of the estimated effects in the remaining effects in the model). Therefore, describing one model (GENS_BMI_) suffices to understand the effect of the covariates in the prediction of diabetes. For GENS_BMI_, gender had an OR = 0.60 which implies a much lower risk of developing T2D in women when compared to men. The Cohort's odds ratio (OR = 0.45), implies a lower risk of T2D in Offspring members in comparison to the Original Cohort. Exposure time had an OR of 1.03, resulting in a 3% increase in risk of development for every year of exposure. The OR for the Genetic Score is approximated to 1.1, which implies an increase in risk of developing T2D, with the increase in value of the genetic score. The OR for BMI was 1.13 in the models that included BMI. This value demonstrates there is a 13% increment in risk of T2D when increasing 1 kg/m^2^ in BMI.

**Table 4:** Estimated odd ratios (95% C.I) for covariates in risk assessment models^**^.

**Covariates**	**BASE**	**BASE_**BMI**_**	**GEN65**	**GEN65_**BMI**_**	**GENS**	**GENS_**BMI**_**
Gender	0.63 (0.54–0.73)	0.61 (0.52–0.71)	0.61 (0.53–0.72)	0.59 (0.51–0.70)	0.62 (0.53–0.72)	0.60 (0.51–0.70)
Cohort	0.52 (0.42–0.64)	0.45 (0.36–0.56)	0.51 (0.40–0.64)	0.45 (0.35–0.57)	0.52 (0.42–0.65)	0.45 (0.36–0.57)
Exposure Time	1.03 (1.02–1.04)	1.04 (1.03–1.05)	1.03 (1.02–1.04)	1.04 (1.03–1.05)	1.03 (1.02–1.04)	1.04 (1.03–1.05)
GS	–	–	–	–	1.07 (1.05–1.08)	1.07 (1.05–1.09)
BMI	–	1.12 (1.11–1.14)	–	1.13 (1.11–1.15)	–	1.13 (1.11–1.14)

** Odds Ratio for the genetic score are only reported for the only two models where it was included.

### SNP estimated effects

Table 5 provides the *P*-value of the 21 SNPs that gave a statistical association with T2D in our study; we also present the *P*-value of those SNPs, in association to BMI and WHR as reported in the Giant Consortium (Heid et al., 2010; Speliotes et al., 2010). Only four SNPs found in the genes GLIS3, PTPRD, TCF7L2, and TSPAN8; had an association with a *P*-value less than 0.001. The SNPs: rs11717195, rs17301514, rs4299828, rs11063069, and rs10842994 have a *P*-value less than 0.1, therefore suggested as possible risk genetic variants. A total of three SNPs, each pertaining to a different gene, were found to be associated to WHR. These genes were: *GCKR* (Glucokinase Regulatory Protein), *IGF2BP2* (Insulin-Like Growth Factor 2 MRNA Binding Protein 2), and *PTPRD* (protein tyrosine phosphatase receptor D). In addition, two SNPs strongly associated to BMI, were located in the genes *IRS1* (Insulin Receptor Substrate 1) and *TCF7L2* (Transcription Factor 7-Like 2).

**Table 5:** ***P*-value for the evaluated SNPs and their reported *P*-values for association to WHR and BMI in the giant consortium**.

**SNP**	**Gene**	***P*-value**	BMI *P*-value^***^	WHR *P*-value^***^
rs780094	*GCKR*	0.0029	0.093	0.00026
rs2943640	*IRS1*	0.0418	0.006	0.60
rs11717195	*ADCY5*	0.0508	0.049	0.10
rs4402960	*IGF2BP2*	0.0131	0.020	0.003
rs17301514	*ADIPOQ*	0.0609	0.155	0.450
rs7756992	*CDKAL1*	0.0337	0.070	0.230
rs4299828	*IRS4*	0.0991	0.474	0.530
rs3734621	*KIF6*	0.0378	0.082	0.190
rs849135	*JAZF1*	0.0418	0.057	0.120
rs10758593	*GLIS3*	0.000532	0.790	0.190
rs16927668	*PTPRD*	0.0012	0.999	0.006
rs10811661	*CDKN2B*	0.0050	0.891	0.110
rs7903146	*TCF7L2*	1.23E-06	0.00024	0.310
rs163184	*KCNQ1*	0.0264	0.887	0.590
rs10830963	*MTNR1B*	0.02918	0.211	0.42
rs11063069	*CCND2*	0.066935	0.127	0.49
rs10842994	*KLHDC5*	0.065763	0.367	0.53
rs7955901	*TSPAN8/ LGR5*	0.000192	0.836	0.18
rs12427353	*HNF1A*	0.02744	0.746	0.61
rs7177055	*HMG20A*	0.014363	0.051	0.23
rs11651052	*TCFL4*	0.008092	–	–

*** P-values of BMI and waist-to-hip ratio (WHR) as reported by GIANT consortium. (Lindgren et al., 2009).

### Interaction with BMI

Our results suggest SNP by BMI interaction with five SNPs at a *P* < 0.05, and 8 genes SNPs with *P* < 0.1. These results along with the estimated OR are provided in Table 6, for all SNPs. The location of the interacting SNPs are in/near the following genes: the Transcription Factor 7 like 2 (*TCFL2*), Gastric Inhibitory Polypeptide Receptor (*GIPR*), Growth Factor Receptor-Bound Protein (*GRB14*), G1/S-Specific Cyclin D2 (*CCND2*), Transducin-Like Enhancer of Split 1 (*TLE1*), Cartilage Intermediate Layer Protein 2 (*CILP2*) and HNF1 homeobox B (*HNF1B*). Genes *CILP2*, *HNF1B*, and *HMGA2*, were confirmed to have an association with BMI (*P* < 0.001). We did not detect any significant interaction in the model where genetic effects were incorporated as a Genetic Score (i.e., GENS_BMI_).

**Table 6:** **Odds Ratio of SNP by BMI interactions of highest significance**.

**SNP**	**Gene**	**Odds Ratio (95%C.I)**	***P*-value**
rs8108269	*GIPR*	1.02 (1.0–1.05)	0.0896
rs13389219	*GRB14*	1.02 (1.00–1.04)	0.0421
rs11063069	*CCND2*	1.02 (0.99–1.05)	0.0870
rs7903146	*TCF7L2*	1.02 (1.00–1.04)	0.0404
rs2796441	*TLE1*	0.97 (0.95–1.00)	0.0231
rs10401969	*CILP2*	1.08 (1.03–1.13)	0.001906
rs11651052	*HNF1B*	0.95 (1.03–1.13)	0.000124
rs2261181	*HMGA2*	0.96 (0.93–0.99)	0.005184

### Predictive ability

Predictive ability of the models was evaluated with a ten-fold cross validation and measured in terms of AUC. Values of the AUC in cross validation, for all risk assessment models in the Logistic Regression and Neural Networks, are reported in Table 7. In addition, ROC Curves for each risk assessment model tested with the Neural Networks, can be found in Table B the Supplementary Material.

**Table 7:** **Predictive ability of the models evaluated with the area under the receiver operating curve (AUC)**.

**Risk assessment models**	**LR**	**NN**
BASE	0.6658	0.6666
BASE_BMI_	0.7393	0.7354
GEN65	0.6785	0.6786
GEN65_BMI_	0.7452	0.7411
GENS	0.6858	0.6857
GENS_BMI_	0.7495	0.7496
GENB_SNPxBMI_	0.7362	0.7432

The AUC of the logistic regression in the BASE model was 0.6658 and 0.666, in the LR and NN models respectively. The incorporation of BMI (BASE_BMI_), increased the AUC to 0.739 and 0.735 for LR and NN, respectively. Also, accounting for genetic markers in GEN65, increased the predictive ability of the models by approximately 2%, when compared to the baseline factors alone. We further analyzed the extent to which the predictive accuracy could be improved by adding BMI to the GEN65 model and achieved a discriminative value of 0.745 and 0.741 (LR and NN, respectively), resulting in an increase of approximately 7%. Previous studies have shown a correlation between the increases in weight and body mass with an increase in probabilities of developing T2D. The incorporation of the genetic score after accounting for BMI further increased AUC to 0.750 (i.e., the GENS_BMI_ model, for both LR and NN). A difference of approximately 8% in predictive ability was observed in the GEN65_BMI_ model, when compared with the baseline model (see Table 7). The inclusion of the interaction of the SNPs with BMI in T2D, gave an AUC of 0.7362 in the GENB_SNPxBMI_ model; with a 0.7% increase when modeled in the Neural Network. Both statistical methods yielded approximately the same AUC. Predictive values show that when strong genetic variants related to T2D are chosen, they substantially improve prediction of risk for T2D.

## Discussion

In this paper we investigated the effects of including genetic information in preventive risk assessments for T2D, while using different modeling approaches (LR and NN). The effect of including genetic information was examined by adding 65 candidate SNPs for T2D and computing a genetic score based on these SNPs.

Of the 65 SNPs analyzed, 7 SNPs that are located in 4 genes (*GLIS3*, *TCF7L2, LGR5*, and *PTPRD)*, showed a strong association with Type 2 Diabetes. In addition, *IGF2BP2* and *GCKR* have been identified by several meta-analyses (Dupuis et al., 2010; Heid et al., 2010; Speliotes et al., 2010; Morris et al., 2012) as risk genetic variants for Type 2 Diabetes with effects in WHR. The SNPs: rs780094, rs7756992, rs7955901 are in the *GCKR*, *CDKAL1*, and *LGR5* gene regions; with annotated functions of insulin production, pancreatic cell growth, and glucose homeostasis, respectively. *GLIS3* has been listed as a diabetes susceptibility gene due to its role in the generation of pancreatic beta cells; an alteration in the expression of this gene could repress the generation of beta cells, and may be involved in pancreatic dysfunction (Dupuis et al., 2010; Nogueira et al., 2013). *TCF7L2* was observed to have a relationship with BMI in both the DIAGRAM and GIANT consortiums (Lindgren et al., 2009; Morris et al., 2012). It has demonstrated to lower insulin secretion by affecting β-cell responsiveness to insulin; it is also found in chromatin regions in islets (Kiessling and Ehrhart-Bornstein, 2006; Sladek et al., 2007; Lyssenko et al., 2008; Mccarthy and Zeggini, 2009). The gene *PTPRD* (protein tyrosine phosphatase receptor type D) provides a component needed to trigger the reactions for the linkage of the insulin receptor to tissue. However, it was excluded as a risk gene for Type 2 Diabetes by Bektas et al. (2001) since none of the mutations did segregate with diabetes. *IRS1* showed an association with BMI through SNP-by-BMI interaction. This genetic variant, with an increased interaction with multiple proteins, has been associated with T2D and obesity, and could lead to the development of insulin resistance (Rung et al., 2009; Caruso et al., 2014).

When analyzing the effects of the inclusion of genetic variants in the prediction of this disease, our results suggest that a vast number of SNPs provide a modest enhancement in the predictive ability of the models. Improvement of these discriminative values, show that the added SNPs capture genetic risk. However, when the interaction of the SNPs by environment (BMI) was included in the model, no further increase was seen. The consistency of AUC throughout the models, with the use of both Neural Network and Logistic Regression, suggests that the use of different statistical approaches neither aided nor reduced the predictive ability of the models. The limitation in predictive accuracy seems to be associated to factors other than the statistical model, such as: the size of the training sample, the number of SNPs included in the model, missing heritability issues and low heritability of the trait. A few concerns about SNPs information, were observed. The first pertains to the imputation uncertainty of the SNPs, since it was not fully taken into account in our analyses. Nevertheless, an alternative methods that consider imputation uncertainty are proposed by Marchini and Howie (2010). Secondly, biases could have been produced in the SNPs estimates due to family structure; nevertheless, since the number of families within our sample is large, it is considered to be of minor importance. In our sample of 5245 subjects, 2073 subjects were aggregated from 495 families, (these families contained subjects with at least one relative in the sample), moreover, the size of these families was 4.19 ± 6.40 (mean ± s.d) members per family.

The most commonly identified covariates used in assessment analyses that provide a high AUC (0.60–0.80) as a clinical baseline model have been: age, high blood pressure, and glucose levels between other covariates (Hu et al., 2001; Lyssenko et al., 2008; Meigs et al., 2008; Cooke et al., 2012). Due to the small effects and marginal change that genotyped data provides in risk prediction, they have been used in only a few models to quantify individual disease risk and thus to facilitate personalized management of T2D risk. The ability and the effects of including genetic information into risk prediction, have been widely studied but are still limited. Previous risk assessments were SNPs associated to T2D were included, slightly improved their predictive ability when compared to baseline clinical covariates (Lyssenko et al., 2008; Meigs et al., 2008; Van Hoek et al., 2008; Katsios, 2010; Bao et al., 2013; Lyssenko and Laakso, 2013; Talmud et al., 2014). In her study, Van Hoek et al. (2008), incorporated 18 SNPs, together with age, sex, and BMI and achieved an AUC of 0.68, yielding only a approximately 2% increase when compared to the baseline model. Furthermore, Lyssenko et al. (2008), evaluated the inclusion of a genetic score built with 16 SNPs; in addition to, multiple clinical covariates and achieved a discriminative value of 0.74. The addition of a modest amount of SNPs into risk prediction was lately studied by Talmud et al. (2014), with the use of 65 SNPs found by the DIAGRAM consortium, which were the same used in this study. A genetic score and clinical covariates such as: BMI, triglyceride levels and fasting glucose, altogether with a large data set, resulted in an AUC of 0.75. This last result is consistent with our results in the model GENS_BMI_. A limitation of our study is that we did not take into account other clinical variables that have shown some degree of association with diabetes, such as triglyceride levels, high blood pressure, LDL or HDL, which could have enhanced our results. The Framingham Heart Study provides these variables, but there are missing values in many exams and subjects. To avoid reducing sample size, we only included BMI longitudinally (i.e., account for BMI at the first diabetes record), and we found that genetic signal from the SNPs is captured beyond what could be explained by the BMI. BMI estimated effect on diabetes may result biased since we incorporated BMI as the BMI at first diabetes diagnosis for diabetic subjects and last BMI on record for healthy subjects. However, preliminary analysis (not included in the paper) show us that the effect and their significance, for BMI and other covariables in the models, are insensitive to alternative ways to account for BMI, such as, BMI at the first exam, or maximum BMI of the subjects observed period. Despite our limitations, our study can provide important remarks. The effect of genetic information in the improvement of the prediction accuracy, was evaluated in our models by incorporating 65 SNPs both directly and into a genetic score. In addition, we looked at the inclusion of gene-environment (BMI) and gene-gene interaction into risk prediction. Also, a classical logistic regression and a Neural Network (a non-parametric classification algorithm) were explored.

Prevalence of T2D is highest among individuals with a BMI ≥ 40 kg/m^2^ (Bays et al., 2007). The increase in central adiposity and percent body fat is associated with an increased risk of T2D; however, not all obese or overweight patients develop T2D, and of those who do, just a proportion is genetically predisposed. Our results show, in agreement with the literature, that BMI serves as a prediction enhancer for T2D. Predictive accuracy yielded better estimates in the baseline model that included BMI; and this was further improved when the genetic effect was also incorporated, giving an AUC difference of a approximately 8% when compared to baseline. Interaction between BMI and the genes: CILP2, HNF1B, and HMGA2 in relation to T2D, was found and reported in Table 6. HNF1B is a homodimer in charge of the nephron and pancreas development. Mutations in this gene region could result in the development of diabetes. In addition, HMGA2 has transcriptional regulating factors which play a role in adipogenesis and fat storage, inducing obesity.

In summary, this study confirmed the association of 21 genetic variants with T2D. It was observed that individuals who have a high genetic score may have increased probabilities of developing Type 2 Diabetes. Also, accounting for genetic information, either by including SNPs or a Genetic Score in the regression, led to an improvement in prediction accuracy (*AUC*) of approximately 2%. However, modeling strategies such as Neural Network or Logistic Regression did not yield differences in terms of prediction. We also showed that the inclusion of BMI into the risk assessment models, improved the predictive accuracy by approximately 8%. Furthermore, the risk assessment model yielded a modest increment in prediction accuracy when including genetic risk score, even after accounting for BMI. This small improvement suggests that there is still genetic signal involved in the development of T2D, yet to be captured, that could produce effects beyond the increase in BMI. In summary, marker information in addition to commonly used baseline covariates such as BMI, could lead to an overall modest improvement of predictive performance.

## Author contributions

All individuals that helped in the writing process of this manuscript are listed as authors and co-authors, and were part of: the formation of the research, recompilation and management of the data, data analysis and interpretation as well as the redaction and edition of this manuscript.

### Conflict of interest statement

The authors declare that the research was conducted in the absence of any commercial or financial relationships that could be construed as a potential conflict of interest.

## Abbreviations

T2D: Type 2 Diabetes
AUC: Area Under the receiver operating Curve
GS: Genetic Score
BMI: Body Mass Index
LR: Logistic Regression
NN: Neural Network
OR: Odds Ratio.
