# Supplementary material for: Integrated genomic and BMI analysis for type 2 diabetes risk assessment
Source: Front Genet. 2015 Mar 17;6:75. doi: 10.3389/fgene.2015.00075 (PMC4362394; doi:10.3389/fgene.2015.00075)
Supplement: Supplementary file 1 [file Table1.DOCX]

***Supplementary Material***

**Integrated genomic and BMI analysis for type 2 diabetes risk assessment.**

**Dayanara Lebrón-Aldea^1^, Emily J. Dhurandhar^2^, Paulino Pérez-Rodríguez^3^, Yann C. Klimentidis^4^, Hemant K. Tiwari ^5^, Ana I. Vazquez^6^.**

^1^Institute of Mathematics, School of Science and Technology, Universidad Metropolitana, San Juan P.R

^2^Department of Health Behavior, School of Public Health, University of Alabama at Birmingham, Birmingham, Alabama

^3^Department of Statistics, Colegio de Postgraduados, Texcoco-México.

^4^Division of Epidemiology and Biostatistics, Mel and Enid Zuckerman College of Public Health, University of Arizona, Tucson, AZ.

^5,6^Department of Biostatistics, School of Public Health, University of Alabama at Birmingham, Birmingham, Alabama, USA.

## Supplementary Tables

Supplementary Table A: IMPUTE2 software results of the imputation accuracies for the 65 SNPs.

| SNP | Accuracy |
| --- | --- |
| rs7903146 | 0.976 |
| rs11257655 | 0.952 |
| rs12242953 | 0.971 |
| rs12571751 | 0.996 |
| rs1111875 | 0.984 |
| rs2334499 | 0.963 |
| rs5215 | 1 |
| rs10923931 | 1 |
| rs163184 | 0.931 |
| rs1552224 | 0.943 |
| rs10830963 | 0.895 |
| rs12427353 | 0.936 |
| rs2075423 | 0.912 |
| rs10842994 | 1 |
| rs11063069 | 1 |
| rs2261181 | 0.997 |
| rs7955901 | 0.992 |
| rs1359790 | 1 |
| rs4502156 | 0.964 |
| rs7177055 | 0.973 |
| rs11634397 | 0.888 |
| rs2007084 | 0.647 |
| rs12899811 | 0.977 |
| rs9936385 | 0.999 |
| rs7202877 | 0.893 |
| rs2447090 | 0.878 |
| rs11651052 | 0.543 |
| rs12970134 | 0.996 |
| rs10401969 | 0.899 |
| rs8182584 | 0.959 |
| rs8108269 | 0.751 |
| rs4812829 | 0.88 |
| rs7569522 | 1 |
| rs13389219 | 0.977 |
| rs2943640 | 1 |
| rs780094 | 1 |
| rs10203174 | 1 |
| rs243088 | 0.978 |
| rs11717195 | 0.984 |
| rs1801282 | 1 |
| rs4402960 | 1 |
| rs17301514 | 0.914 |
| rs1496653 | 1 |
| rs12497268 | 0.937 |
| rs6795735 | 1 |
| rs6819243 | 0.963 |
| rs4458523 | 1 |
| rs459193 | 0.986 |
| rs6878122 | 0.73 |
| rs7756992 | 0.968 |
| rs4299828 | 1 |
| rs3734621 | 0.936 |
| rs17867832 | 0.95 |
| rs13233731 | 1 |
| rs17168486 | 0.939 |
| rs849135 | 0.987 |
| rs10278336 | 0.833 |
| rs3802177 | 0.974 |
| rs516946 | 0.988 |
| rs7845219 | 0.994 |
| rs10811661 | 1 |
| rs10758593 | 0.986 |
| rs17791513 | 0.963 |
| rs16927668 | 0.939 |
| rs2796441 | 0.723 |

Supplementary Table B. ROC curves for models examined with the Neural Network.

| Model | ROC | AUC |
| --- | --- | --- |
| BASE | 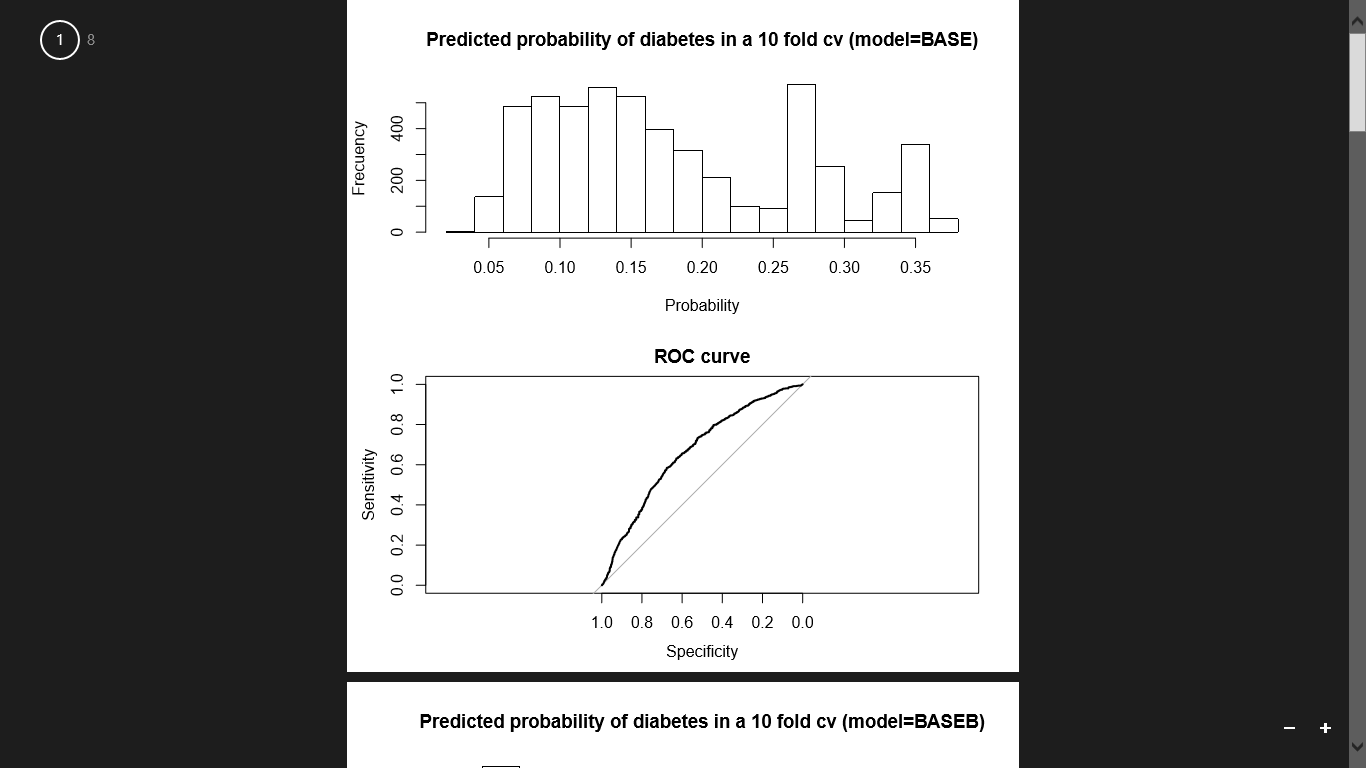 | 0.6666 |
| BASE_BMI_ | 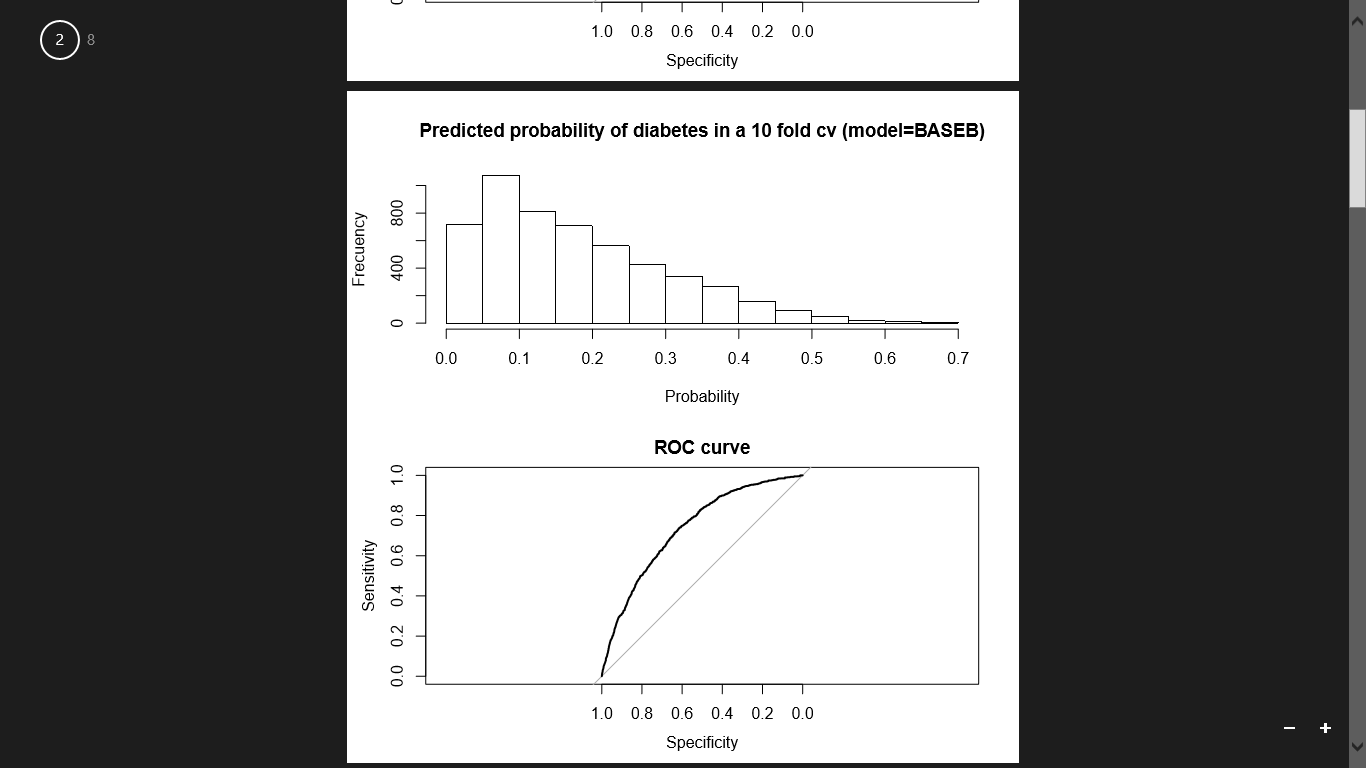 | 0.7354 |
| GEN65 | 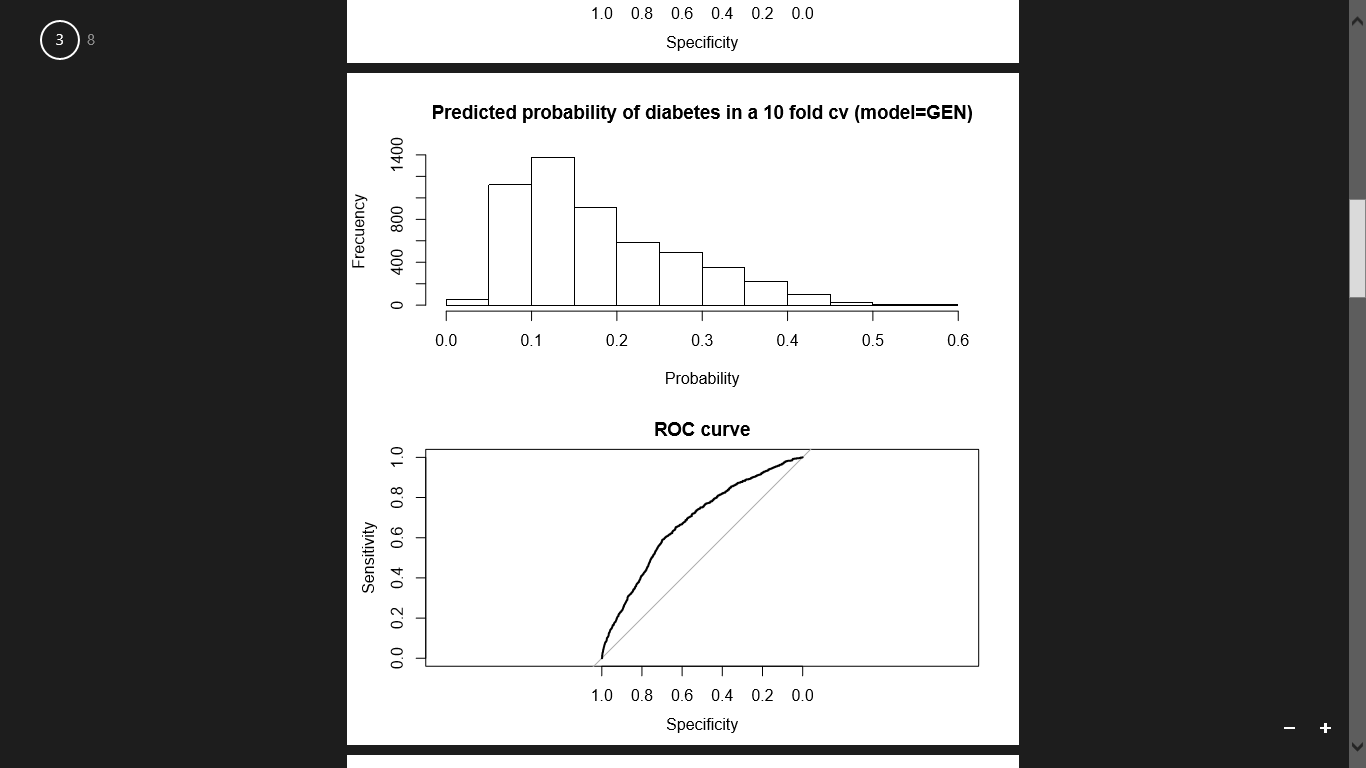 | 0.6786 |
| GEN65_BMI_ | 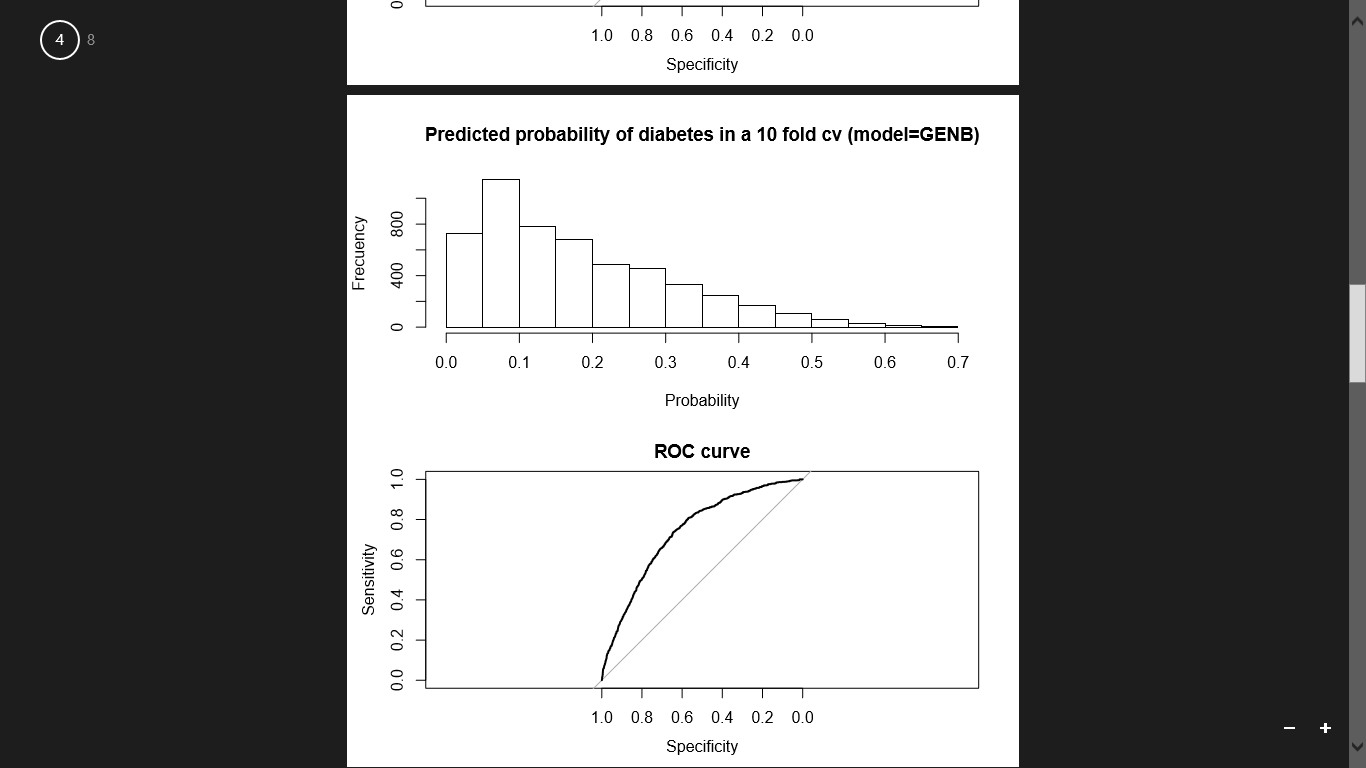 | 0.7411 |
| GENS | 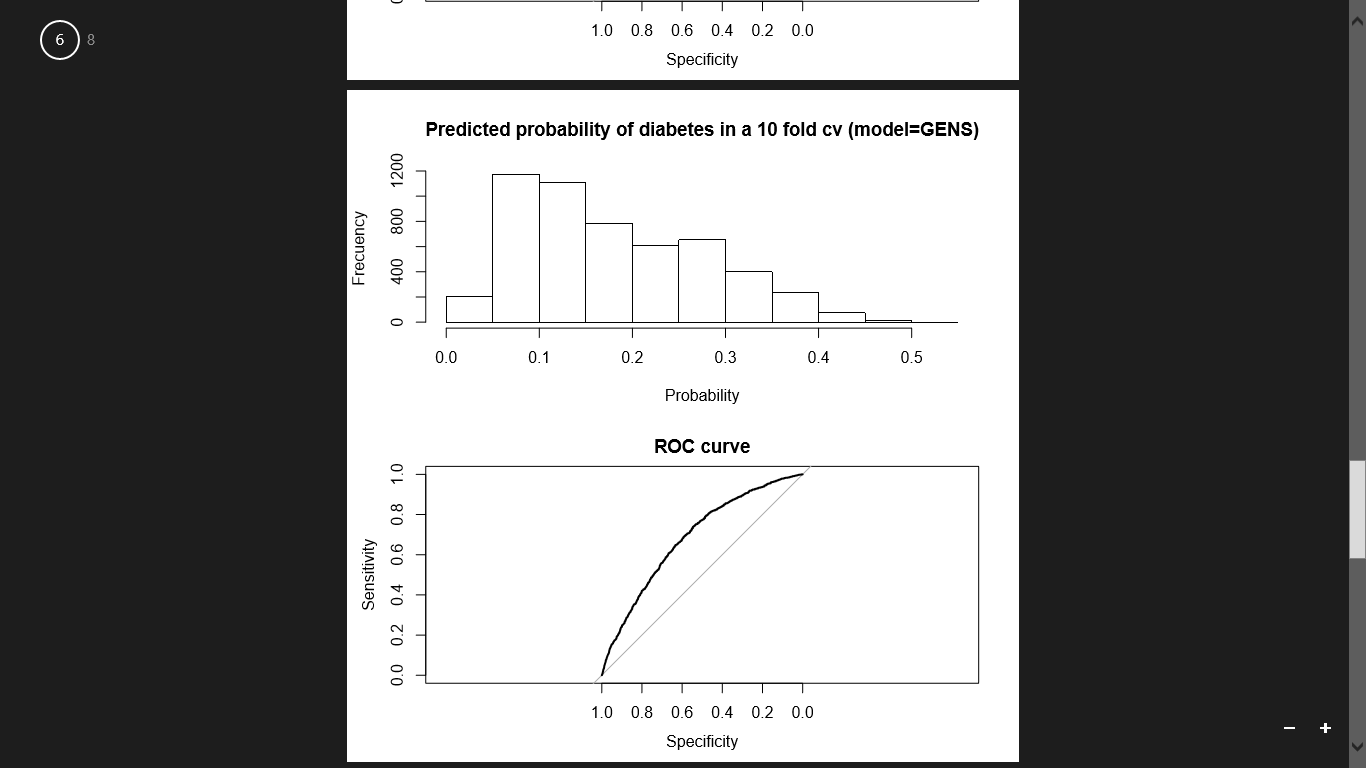 | 0.6857 |
| GENS_BMI_ | 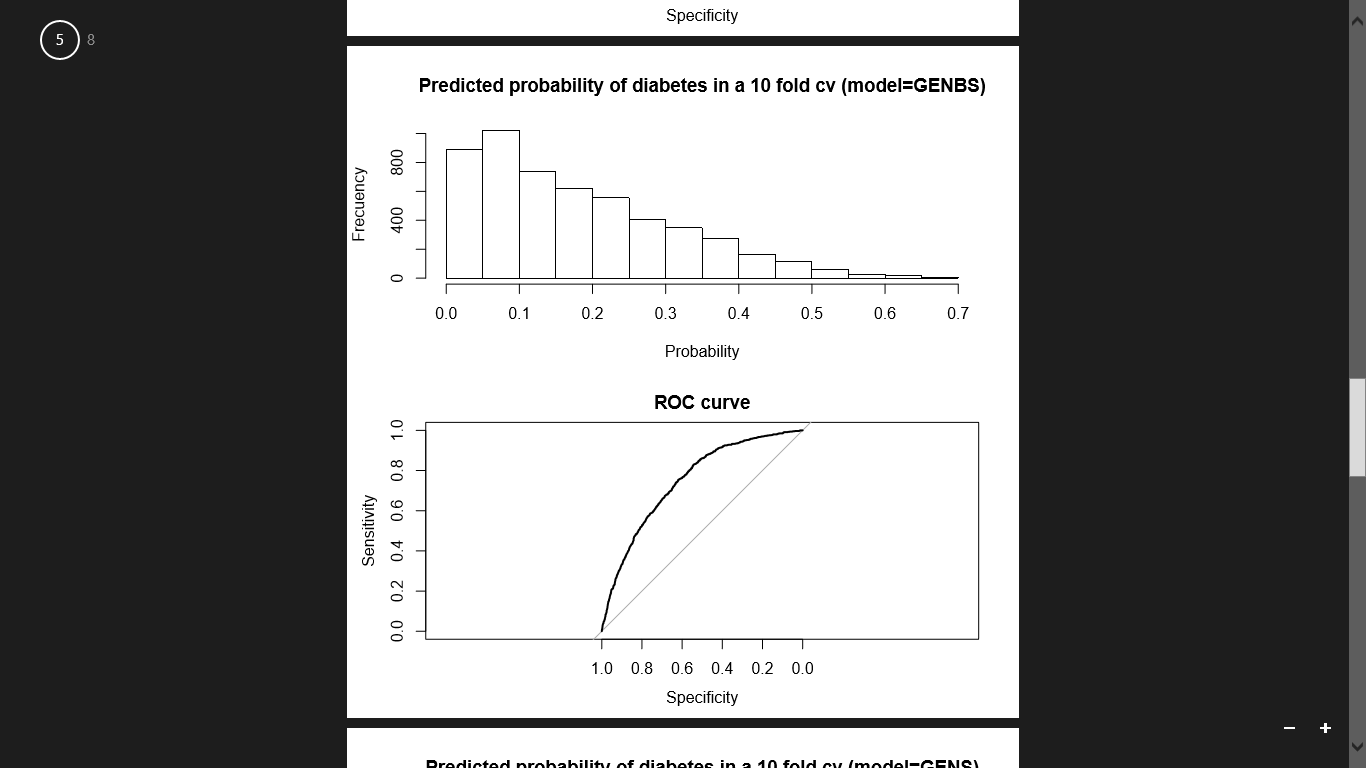 | 0.7496 |
| GENB_SNPxBMI_ | 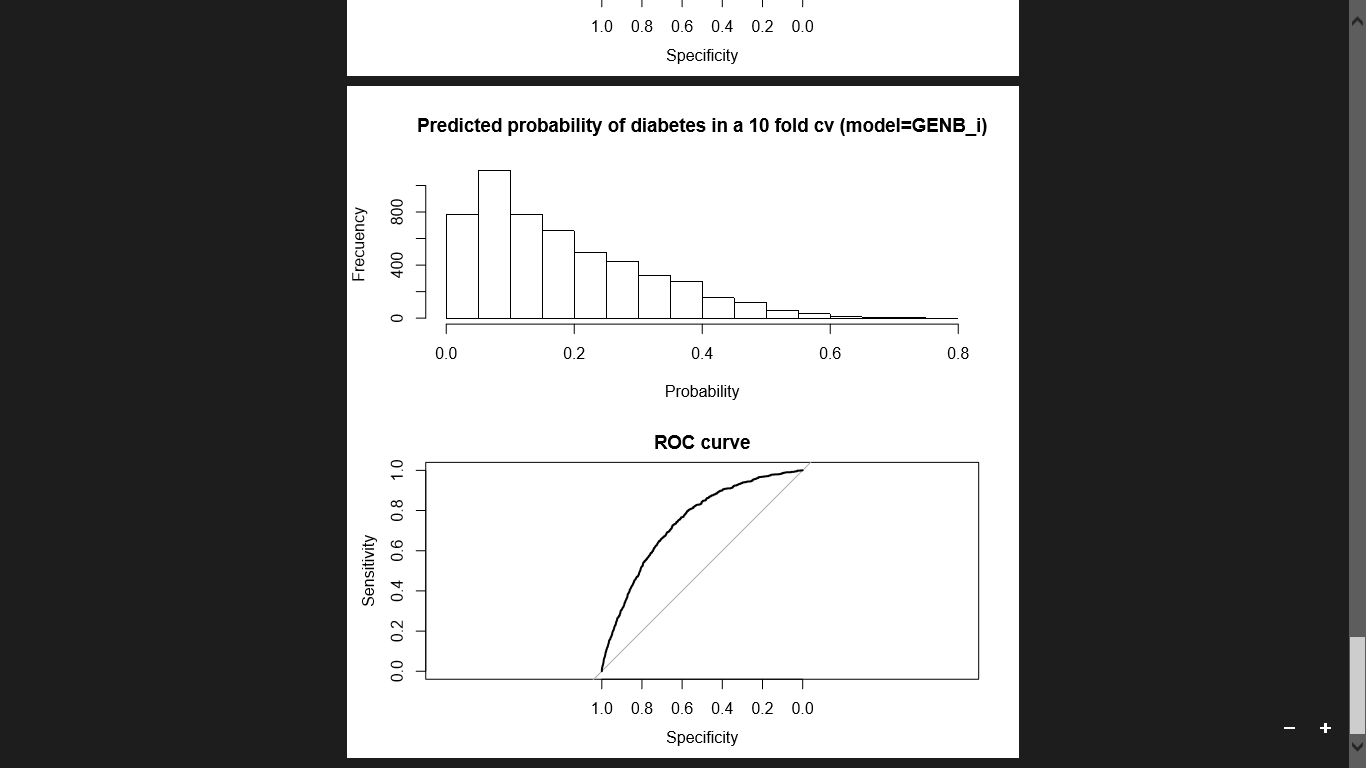 | 0.7432 |
